# CDC42 Negatively Regulates Testis-Specific SEPT12 Polymerization

**DOI:** 10.3390/ijms19092627

**Published:** 2018-09-05

**Authors:** Chia-Yen Huang, Ya-Yun Wang, Ying-Liang Chen, Mei-Feng Chen, Han-Sun Chiang, Pao-Lin Kuo, Ying-Hung Lin

**Affiliations:** 1 Department of Biological Science and Technology, National Chiao Tung University, Hsinchu 300, Taiwan; bagiao2003@gmail.com; 2 Gynecologic Cancer Center, Department of Obstetrics and Gynecology, Cathay General Hospital, Taipei 106, Taiwan; 3 School of Medicine, Fu Jen Catholic University, New Taipei City 242, Taiwan; 4 Department of Chemistry, Fu Jen Catholic University, New Taipei City 242, Taiwan; vic0009@gmail.com; 5 Department of Environmental Engineering, National Cheng Kung University, Tainan 701, Taiwan; roy.yl.chen@gmail.com; 6 Bone and Joint Research Center, Chang Gung Memorial Hospital, Taoyuan 333, Taiwan; mfchen0@gmail.com; 7 Graduate Institute of Biomedical and Pharmaceutical Science, Fu Jen Catholic University, New Taipei City 242, Taiwan; 053824@mail.fju.edu.tw; 8 Department of Obstetrics & Gynecology, College of Medicine, National Cheng Kung University, Tainan 701, Taiwan; paolink@mail.ncku.edu.tw

**Keywords:** SEPT, SEPT12, CDC42, sperm

## Abstract

*Septin* (*SEPT*) genes encode well-preserved polymerizing GTP-binding cytoskeletal proteins. The cellular functions of SEPTs consist of mitosis, cytoskeletal remodeling, cell polarity, and vesicle trafficking through interactions with various types of cytoskeletons. We discovered that mutated *SEPTIN12* in different codons resulted in teratozoospermia or oligozoospermia. In mouse models with a defective *Septin12* allele, sperm morphology was abnormal, sperm count decreased, and sperms were immotile. However, the regulators of SEPT12 are completely unknown. Some studies have indicated that CDC42 negatively regulates the polymerization of SEPT2/6/7 complexes in mammalian cell lines. In this study, we investigated whether CDC42 modulates SEPT12 polymerization and is involved in the terminal differentiation of male germ cells. First, through scanning electron microscopy analysis, we determined that the loss of *Septin12* caused defective sperm heads. This indicated that *Septin12* is critical for the formation of sperm heads. Second, CDC42 and SEPT12 were similarly localized in the perinuclear regions of the manchette at the head of elongating spermatids, neck region of elongated spermatids, and midpiece of mature spermatozoa. Third, wild-type CDC42 and CDC42Q61L (a constitutive-acting-mutant) substantially repressed SEPT12 polymerization, but CDC42T17N (a dominant-negative-acting mutant) did not, as evident through ectopic expression analysis. We concluded that CDC42 negatively regulates SEPT12 polymerization and is involved in terminal structure formation of sperm heads.

## 1. Introduction

### 1.1. Septin Family

Septins (SEPTs) are the fourth component of the cytoskeleton—the others are actin filament, microtubule, and intermediate filaments—and are an evolutionarily conserved family of polymerizing GTP-binding proteins [1]. SEPTs were first identified as *cell division cycle* (*cdc*) mutants of the four *Septin* genes, *cdc3*, *cdc10*, *cdc11*, and *cdc12*, in *Saccharomyces cerevisiae*. SEPTs assemble into concentric filaments at the mother-bud neck during cell division, forming a diffusion barrier, and loss-of-function mutants of any of the four *Septin* genes lead to multinucleated cells with defective division [2,3]. SEPTs participate in many cellular functions such as cytokinesis, membrane dynamics, compartmentalization, vesicle trafficking, cell polarity determination, cytoskeletal remodeling, and apoptosis through interaction with several types of cytoskeletal proteins (e.g., tubulins, actin, and myosin II) [1,4,5]. SEPT complexes, such as SEPT2/6/7 and SEPT1/4/6/7 complexes, form filament-like structures in cells, thus facilitating various cellular functions [6,7]. Thus far, 14 classes of *SEPTs* have been recognized in mammalian cells, and several *SEPTs* are specifically expressed in well-differentiated cells (e.g., neurons or male germ cells), whereas the others are generally expressed [5].

### 1.2. Functional Roles of SEPTs in Mammalian Spermatogenesis

SEPT4 plays a critical role in maintaining the correct structure of the midpiece of the flagellum and annulus, which is a ring-like structure between the midpiece and the principal piece of the flagellum [7,8]. Defective sperms isolated from *Sept4* knockout male mice were related to infertility because the damaged annulus and midpiece caused sperm immobility. Clinically, SEPTs (SEPT1/4/6/7 complexes) were determined to be lacking in a high proportion of asthenozoospermia cases [7,9]. In our previous studies, the testicular tissues of infertile and fertile men were compared using cDNA microarray analysis, which indicated that *SEPT12* is a potential sterility-related gene [10]. Moreover, sperm cells isolated from *Septin12* knockout mice exhibited distinctive morphological defects: defective sperm heads, bent tails, premature chromosomal condensation, and nuclear damage [11]. And, SEPT12 is restricted to and localized in postmeiotic male germ cells, forms the filamentous structure around the manchette structure of elongating spermatids. The filamentous structure was similar to that of the overexpressed *SEPT12* in Chinese hamster ovary (CHO), Hela, and 293T cells [11,12,13]. Mutations of SEPT12 have been determined to cause teratozoospermia and oligozoospermia [14,15].

### 1.3. CDC42 and SEPTs

Filament-like polymer constitutes the major functional structure of SEPTs [1,16]. Some studies have identified that posttranslational modification of SEPTs and CDC42 and its effectors regulate the dynamics and assembly/disassembly of the SEPT polymer in yeasts [2,17]. In budding yeasts, CDC42 regulates SEPT ring assembly, but not Rho, at the bud neck [18,19]. In mammalian cells, the assembly of SEPTs (SEPT2/6/7) is negatively regulated by CDC42, which inhibits the association of BORG (Binder of Rho GTPases), a downstream effector of CDC42, with SEPT2/6/7 [20,21]. Furthermore, immunohistochemical analysis revealed that the expression signals of CDC42 in testicular sections were the most intense signals surrounding the elongated spermatids [22]; this is similar to the expression patterns of SEPT12 in testicular sections [23].

In this study, we investigated whether CDC42 also regulates SEPT12 and is involved in the terminal differentiation of male germ cells.

## 2. Results

### 2.1. Using Scanning Electron Microscopy to Evaluate Sperm Heads from Septin12^+/−^ Adult Mice

**Figure 1:**
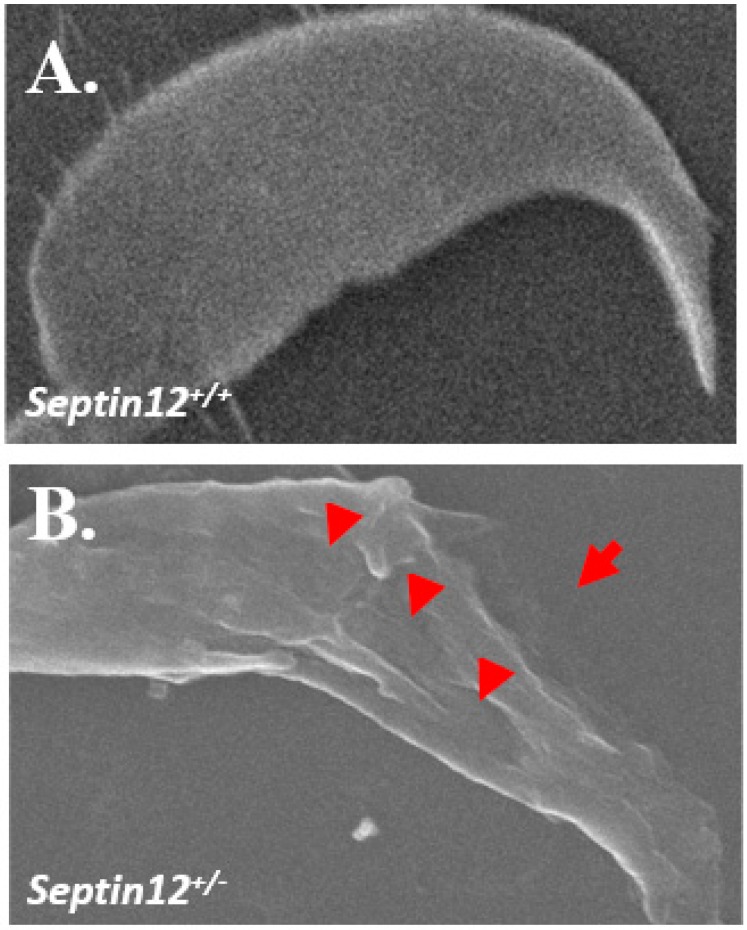
Spermatozoa from *Septin12* knockout mice with abnormal head shapes. Scanning electron microscopy (SEM) images of sperm cells isolated from (**A**) *Septin12*^+/+^ and (**B**) *Septin12*^+/−^ mice. Arrowheads indicate abnormalities on the outside membrane of the sperm head. The arrow indicates the disrupted acrosome (Magnification: ×3000).

Numerous morphological defects have been detected in sperm from *Septin12*^+/−^ mice through chemical staining [24]. However, details concerning the morphological status of sperm heads remain unknown. Scanning electron microscopy (SEM) was used to precisely evaluate the integrity of sperm heads. The head structure of mature sperm from *Septin12*^+/+^ mice exhibited a completed and sharp hook-like structure (Figure 1A). Observed through SEM and compared with wild-type sperm cells, sperm from *Septin12*^+/−^ mice exhibited severely disrupted sperm head patterns at the acrosome (arrow indicated) and covering membrane (arrowhead indicated; Figure 1B). We discovered that the loss of *Septin12* damaged the structural integrity of the sperm heads, which is critical for maintaining the sperm nuclei.

### 2.2. Dynamic Expression Patterns of CDC42 during Murine Spermiogenesis

**Figure 2:**
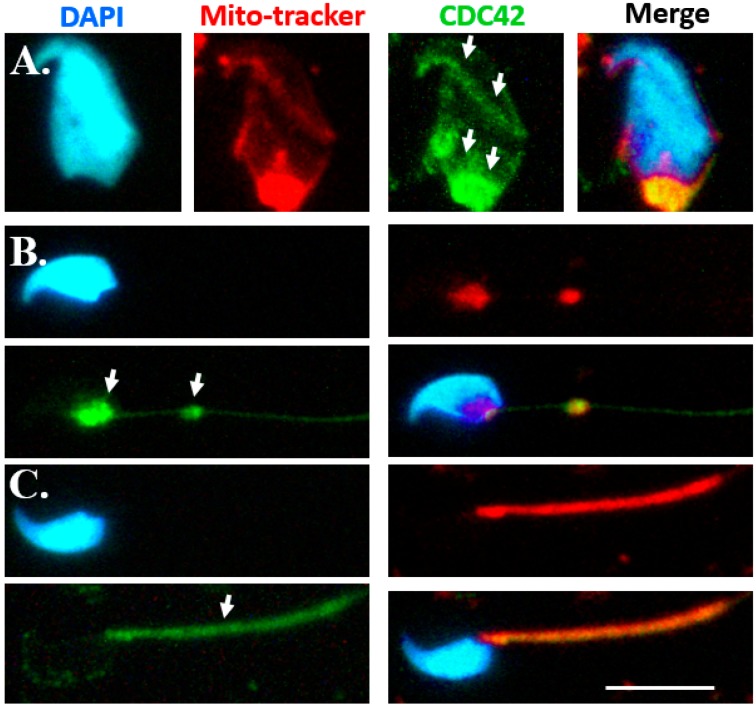
CDC42 localization during murine spermiogenesis. Immunofluorescence (IF) staining results revealed multiple localizations of CDC42 signals: (**A**) elongating spermatids, (**B**) elongated spermatids, and (**C**) mature spermatozoa. Nuclear staining (“DAPI” panels), mitochondria labeling (“Mitotracker”), anti-CDC42 antibody (“CDC42” panels), and a combination of DAPI, Mitotracker, and anti-CDC42 antibody (“Merge” panels) were used as stains. Arrowheads indicate CDC42 localizations. Scale bar = 10 μm.

**Figure 3:**
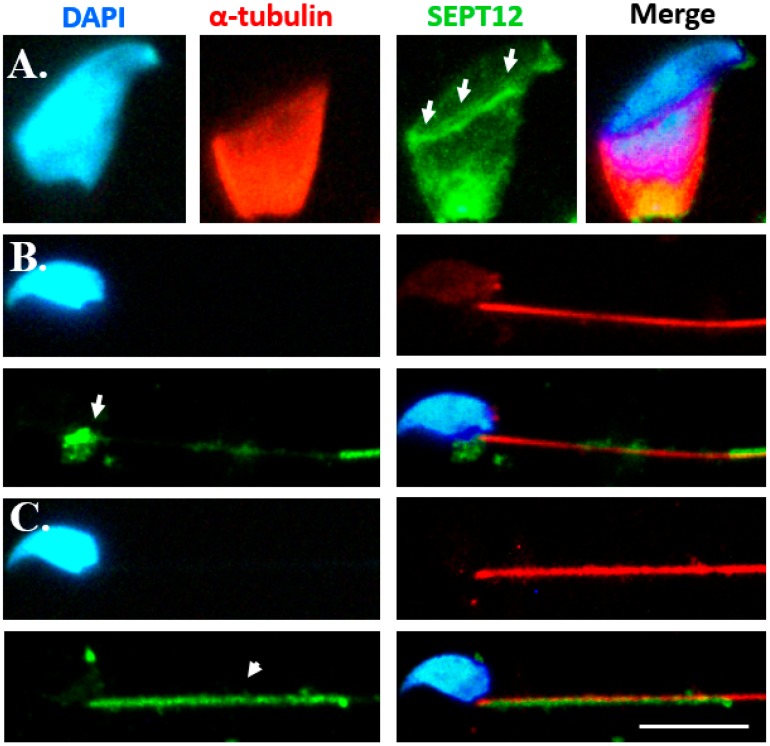
SEPT12 localization during murine spermiogenesis. IF staining results revealed multiple localizations of SEPT12 signals: (**A**) elongating spermatids, (**B**) elongated spermatids, and (**C**) mature spermatozoa. DAPI (“DAPI” panels), anti-α-tubulin antibody (“α-tubulin” panels), anti-SEPT12 antibody (“SEPT12” panels), and a combination of DAPI, anti-α-tubulin antibody, and anti-SEPT12 antibody (“Merge” panels) were used as stains. Arrowheads indicate SEPT12 expression. Scale bar = 10 μm.

Several studies have indicated that CDC42 regulates the polymerization of SEPTs in yeast and mammalian cell lines [18,20,21]. However, the detailed dynamic expression patterns of CDC42 during mammalian spermiogenesis remain unknown. To determine the localization of CDC42 during murine spermiogenesis, the testicular germ cell populations were separated and subjected to immunofluorescence staining. During sperm head formation, CDC42 concentrates around the acrosome, the perinuclear mantle of the manchette structure, and the sperm neck, as depicted in Figure 2A. With the formation of mitochondria, CDC42 is localized at the neck and the mitochondria materials (Figure 2B). Furthermore, in mature spermatozoa, CDC42 is localized in the midpiece region, as depicted in Figure 2C. Localization of CDC42 is similar to SEPT12 localization patterns during murine spermiogenesis in elongating spermatids, elongated spermatids, and mature sperm cells (Figure 3).

### 2.3. CDC42 Alters SEPT12 Polymerization

**Figure 4:**
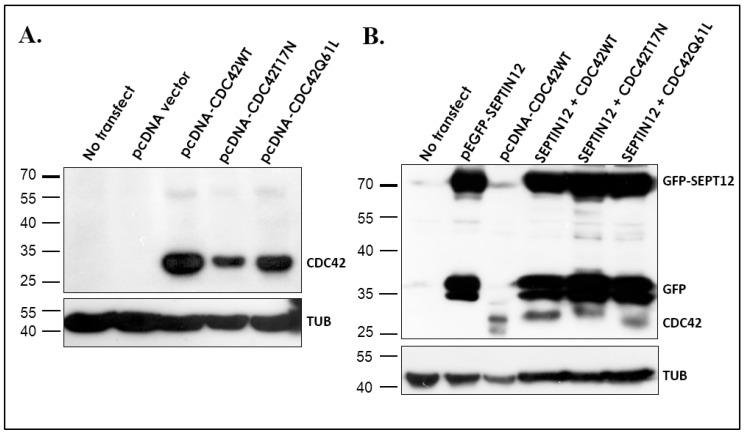
CDC42 and SEPT12 constructs expressed in 293T cells. (**A**) The lysates from cells transfected with mock (no transfect; lane 1), empty pcDNA vector (pcDNA vector; lane 2), pcDNA-Cdc42 wild-type vector (pcDNA-Cdc42WT; lane 3), pcDNA-Cdc42T17N vector (lane 4), or pcDNA-Cdc42Q61L vector (lane 5) were blotted with anti-CDC42 antibody (upper panel), and anti-α-tubulin antibody (bottom panel), as loading controls. (**B**) Lysates from cells transfected with mock (no transfect; lane 1), pEGFP-Sept12 vector (lane 2), pcDNA-Cdc42 wild-type vector (pcDNA-Cdc42WT; lane 3), pEGFP-Septin12 with pcDNA-CDC42WT vectors (SEPTIN12 + CDC42; lane 4), pEGFP-Septin12 with pcDNA-CDC42T17N vectors (SEPTIN12 + CDC42T17N; lane 5), or pEGFP-Septin12 with pcDNA-CDC42Q61L vectors (SEPTIN12 + CDC42Q61L; lane 6). Membranes were blotted with anti-EGFP antibody (GFP-SEPT12 and GFP; upper panel), anti-CDC42 antibody (CDC42; upper panel), and anti-α-tubulin antibody (bottom panel) as loading controls.

**Figure 5:**
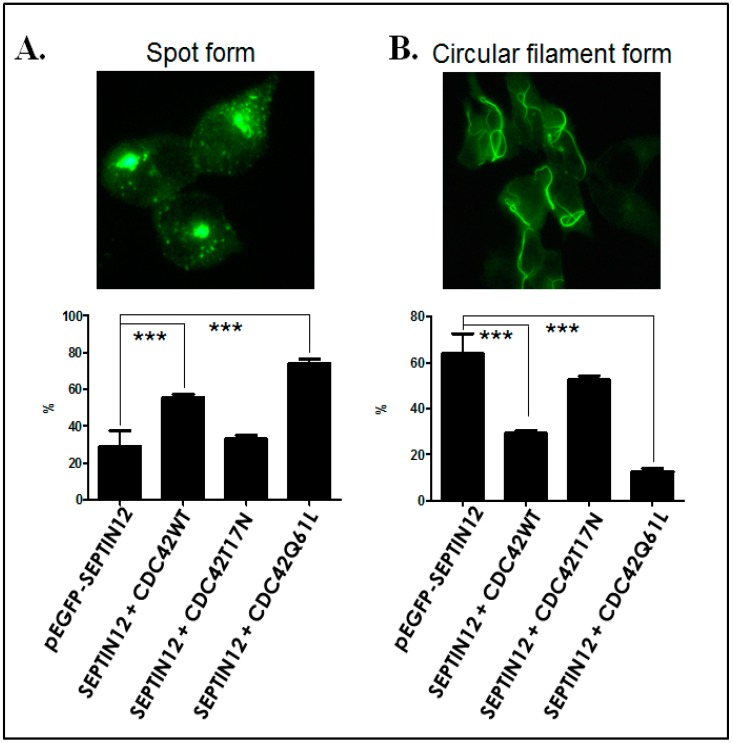
CDC42 overexpression affects SEPT12 polymerization in 293T cells. SEPT12 overexpression leads to a spot form (**A**, upper panel) or a circular filament form (**B**, bottom panel). Magnification: ×400 in (**A**,**B**). (**A**,**B**; bottom panel) Quantification of the percentage of spot and circular filament forms in the transfected cells. The height of the boxes represents the mean of the values obtained from four independent experiments. At least 100 transfected cells were counted in each experiment (*** *p* < 0.001, Student’s *t* test).

Although the expression patterns of CDC42 and SEPT12 were comparable during murine spermiogenesis, whether CDC42 regulates SEPT12 polymerization remains unknown. To investigate, CDC42 (wild type), CDC42T17N dominant-negative-acting mutant, and CDC42Q61L constitutive-acting mutant were first constructed into the pcDNA3.1 vector. The vectors were transfected into 293T cells and verified through western blotting (Figure 4A). Different CDC42 vectors were cotransfected with the pEGFP-Septin12 vector to evaluate the effects. Figure 4B shows that the various CDC42 vectors were coexpressed with the pEGFP-SEPTIN12 vector in 293T cells. SEPT12 overexpression alone exhibited a small percentage of spot form (average: 30.40%, quadruplication) and a large percentage of circular filament form (average: 62.15%, quadruplication; Figure 5). SEPT12 coexpressed with the wild-type CDC42 and CDC42Q61L (constitutively acting) vectors decreased the percentage of circular filament form (average: 29.56%, quadruplication; 12.26%, quadruplication, respectively), thereby substantially increasing the percentage of spot form (average: 55.07%, quadruplication; 74.90%, quadruplication, respectively), as determined through IF staining and cells counts. Dominant-negative-acting CDC42T17N maintained a high percentage of polymerized SEPT12 (circular filament form; average: 52.56%, quadruplication), which was comparable with cotransfected CDC42WT. These results indicated that CDC42 negatively regulated SEPT12 polymerization. These results also demonstrated that CDC42 was involved in modulating SEPT12 polymerization and murine spermiogenesis.

## 3. Discussion

In this study, we discovered that depleted *Septin12* disturbed the structure of the sperm heads in vivo, and localization of CDC42 and SEPT12 appeared similar during sperm head formation. Furthermore, CDC42 was identified as a negative regulator of SEPT12 polymerization. This is the first study to link testis-specific SEPT12 polymerization with CDC42, which is involved in the terminal differentiation of male germ cells.

### 3.1. Loss of Septin12/SEPTIN12 Damages the Structure of Sperm Heads

In *Septin12* knockout mice, SEM revealed a disrupted structure of the sperm heads (Figure 1). This result is consistent with SEPT12 expression patterns at the spermatid head (Figure 3A). During murine spermiogenesis, SEPT12 forms the perinuclear ring of the manchette, which is constituted by microtubules [25,26]. As the development continues, SEPT12 concentrates at the elongating tail and participates in the tail formation. Moreover, it is reminiscent of cases in which *SEPT12* mutated (c.474G/A), induced the truncated SEPT12, and yielded a high percentage of irregular sperm-heads and loss of nuclear materials [15]. This mutation of *SEPT12* is also harmful to its polymerization function, as determined by cell model analysis. We presume that the loss of *Septin12* decreases its polymerization ability, thus leading to teratozoospermia by disturbing sperm head formation.

### 3.2. Dynamic Expression of CDC42 and SEPT12 during Mammalian Spermiogenesis

Some studies have indicated that CDC42 negatively regulates SEPT2/6/7 polymerization in mammalian cells by breaking the binding activity between BORG and SEPT2/6/7 [20,21]. Furthermore, in testicular sections, CDC42 was expressed in the head of spermatids [22]. However, the precise localization of CDC42 during murine spermiogenesis was unknown. In this study, we used separation of male germ cells and the IF assay to determine that CDC42 was expressed in the perinuclear mantle of the manchette structure during sperm head formation and in the midpiece region in mature spermatozoa (Figure 2). The results indicated that CDC42 was involved in sperm head and tail formation. Results also indicated that this was similar to SEPT12 localization during the formation of sperm heads and tails.

### 3.3. CDC42 Alters SEPT12 Polymerization

Most studies evaluating whether CDC42 regulates the polymerization of SEPTs (SEPT2/6/7) have examined mammalian cell lines and cancer cells [20,21,27,28]. Only a few studies have examined the possible regulatory mechanism of CDC42 and SEPTs in vivo, including the brain and spine [29,30]. In a cell model, CDC42 was found to negatively regulate SEPT2/6/7 polymerization in MDCK cell lines [20,21]. In this study, we tried to link the relevant regulatory mechanism of CDC42 to SEPT12 polymerization during sperm formation (Figure 2 and Figure 3). Because of the lack of differential models of male germ cells, we chose 293T cells for further study. We found SEPT12 overexpression revealed lead to the filament form in 293T cells (Figure 5B), which is similar to the SEPT12 filament around the perinuclear mantle of the manchette (Figure 3A). And, CDC42 is a negative regulator for the formation of the SEPT12 filament. 

### 3.4. CDC42/BROG/SEPTs and Terminal Differentiation of Sperm

In mammalian cells, BROG (Binder of Rho GTPases), a downstream effector of CDC42, binds with SEPT2/6/7 and CDC42 [20,31]. Expression of BROG3 binds to SEPT6 and 7 and induces the formation of long and thick SEPT2/6/7 filaments [20]. The localization of BROG3 is distributed with SEPTs, and overexpression of BROG3 appears to bundle or extend the SEPTs filaments. Furthermore, an activated CDC42 mutant binds to BROG and inhibits the binding of BORG3 to SEPT7 in a dose-dependent manner through coimmunoprecipitation. SEPTs redistribute in aggregated patterns after CDC42 is overexpressed. In this study, the terminal differentiations of male germ cells entail a more complicated and dynamic process than cell models. First, at spermatids, enriched SEPT12 and slightly expressed CDC42 are localized at the perinuclear mantle of the manchette (Figure 2A and Figure 3A). In Figure 2A, the highly expressed CDC42 were aggregated at the bottom regions of the spermatid head. SEPT12 was expressed in comparable patterns (Figure 3A), similar to the cell models in which overexpressed CDC42 induced the aggregation of SEPTs. The highly expressed CDC42 and aggregated SEPT12 were localized at the neck region of elongated spermatids and at the midpiece of mature sperm (Figure 2B,C and Figure 3B,C). The dynamic changes of SEPT12 polymerization seemed to be regulated by the negative activation of CDC42.

## 4. Experimental Section

### 4.1. Scanning Electron Microscopy

The animal studies were all approved by the Animal Care Review Board of National Cheng-Kung University Medical College (code: 99087, 14 December 2009). Mature spermatozoa were flushed from the vas deferens of adult male mice and washed with 1× phosphate-buffered saline three times. The suspensions were then centrifuged with maximal force for 10 min, spread on a slide, and air dried. The SEM examination was conducted according to a previously described protocol [32,33].

### 4.2. Testicular Germ Cell Isolation

Separation of spermatogenic cells according to the density of various types of germ cells was performed using a centrifugal system as described previously [34]. After decapsulation and digestion using an enzyme, germ cell suspensions were filtered through 35-μM nylon filters (Falcon, Austin, TX, USA) and then centrifuged. Male germ cells in different developmental stages were collected. Mature spermatozoa were collected from the cauda epididymis of adult male mice. Finally, the suspensions were centrifuged with maximal force for 10 min, spread on a slide, and air dried.

### 4.3. Immunofluorescence Staining

Male germ cells were treated with 0.1% Triton X-100, washed twice with Tris-buffered saline (TBS), and subsequently incubated with a primary antibody (CDC42: Sigma, St. Louis, MO, USA; SEPT12: Abnova, Taipei, Taiwan; α-tubulin: Sigma; Green fluorescent protein (GFP): Santa Cruz, CA, USA) for 60 min at room temperature. After being washed with Tris-buffered Saline (TBS), the sections were incubated with the secondary antibody for 60 min at room temperature and washed again with TBS. A MitoTracker dye (Invitrogen, Waltham, MA, USA) and 4′,6-diamidino-2-phenylindole (DAPI; Invitrogen) were used to stain the mitochondria and nuclei, respectively. Labeled spermatozoa were examined, and images were captured using the upright BX60 microscopy system (Olympus, Tokyo, Japan).

### 4.4. Cloning, Transfection, and Western Blotting

From a human RNA panel, the full lengths of human *SEPTIN12* and *CDC42* were amplified using the reverse transcription polymerase chain reaction technique and cloned into the pEGFP-N1 and pcDNA 3.1 vectors, as described previously [35]. The constructs were confirmed through Sanger sequencing. A CDC42 mutation was prepared using QuickChange Site-directed Mutagenesis Kits (Stratagene, LaJolla, CA, USA). All constructs were confirmed through DNA sequencing. After the cell line was transfected with plasmids using Lipofectamine 2000 (Invitrogen), the cells were subjected to immunoblotting and immunofluorescence (IF) staining. Western blot analysis was performed according to the standard protocol [14].

## 5. Conclusions

In this study, we demonstrated for the first time that CDC42 is a regulator of SEPT12 polymerization and that CDC42/SEPT12 is involved in the terminal differentiation of male germ cells.
